# Effect of the H1N1 Influenza Pandemic on the Incidence of Epidemic Keratoconjunctivitis and on Hygiene Behavior: A Cross-Sectional Study

**DOI:** 10.1371/journal.pone.0023444

**Published:** 2011-08-16

**Authors:** Hyun Su Kim, Ho Chun Choi, Belong Cho, Joon Yong Lee, Min Jeong Kwon

**Affiliations:** 1 Department of Family Medicine, Seoul National University Hospital, Seoul, Republic of Korea; 2 Department of Family Medicine, Korea University Guro Hospital, Seoul, Republic of Korea; 3 Division of Infectious Disease Surveillance, Korea Centers for Disease Control and Prevention, Chungcheongbuk-Do, Republic of Korea; Kenya Medical Research Institute - Wellcome Trust Research Programme, Kenya

## Abstract

**Background:**

EKC is transmitted chiefly by direct hand contact. It is suspected that the 2009/2010 influenza pandemic influenced hand washing. This study aims to examine the relationship between the 2009/2010 H1N1 influenza pandemic and hygiene behavior.

**Methods:**

We compared the EKC prevalence trends before, during and after the 2009/2010 influenza pandemic by using a t-test comparison of EKC sentinel surveillance.

**Results:**

During the pre-pandemic period, the incidence of EKC increased from the 21st to the 44th week each year. However, during the pandemic period in 2009, there was no epidemic peak. In the post-pandemic period, the epidemic curve was similar to that in the pre-pandemic period. Compared to the pre-pandemic period, the total number of EKC patients during the pandemic period showed a decrease of 44.9% (t value = −7.23, p = 0.002). Comparing the pre-pandemic and pandemic periods by age group, we found there to be a significant decrease in the number of EKC patients for all age groups (−4.12≤t value≤−7.23, all P<0.05). This finding was most evident in the teenage group (62%) compared to the other age groups (decreases of 29 to 44%).

**Conclusions:**

A continuing effort should be made to educate the public on basic infection prevention behaviors in the aftermath of the pandemic, particularly to teenagers.

## Introduction

While the World Health Organization (WHO) declared on August 10, 2010 that the influenza A (H1N1) pandemic was over, the Korean Government had already changed the National Infectious Disease Crisis Level to its lowest grading on April 1, 2010 [1], [2]. In Korea, from the time the first case of influenza A was confirmed on May 3, 2009, until late January, 2010, a total of 740,835 patients were confirmed, with 225 of these reported to have died [3]. During the pandemic in Korea, before an effective vaccine was made available, mitigation strategies were aimed at identifying, isolating and treating individual patients, in addition to educating the public about preventive behaviors that could reduce the spread of infection. These messages emphasized covering the mouth and nose when coughing and sneezing, washing hands frequently with soap and water, and avoiding crowded places [4].

It is suspected that the 2009/2010 influenza pandemic influenced hygiene-related behavior such as hand washing. However, previous studies were mainly interested in the association between these behaviors and disease anxiety, flu severity, information reliability and effectiveness of control measures [5]–[11]. While some studies show that these behaviors can be improved transiently [11]–[14], we have little information on how long these improved behaviors will continue or what kind of groups could be most motivated.

Among the various infectious diseases, we considered those that can be transmitted by direct hand contact. As a result, EKC was selected for evaluation. This condition is transmitted chiefly by direct hand contact rather than other pathways, and cannot be prevented by vaccination [15]–[18]. In the present study, we assessed the effect of the 2009/2010 influenza pandemic on these behaviors by analyzing the incidence of this disease before, during and after the influenza pandemic.

## Methods

We analyzed the EKC data of the Korean National Infectious Disease Surveillance System. The system in place to report EKC cases was not affected by the 2009 pandemic season because ophthalmology clinics were not busy managing patients who were infected with influenza. In addition, there were sufficient cases of EKC for analysis.

The sentinel surveillance of EKC is supported by the Korean Ophthalmologists Association and Korean Ophthalmological Society. 80 private ophthalmological clinics voluntarily report cases of patients with EKC on a weekly basis. We used the diagnostic criteria for EKC that were defined for the National Infectious Disease Surveillance. The criteria has been defined as a clinically compatible illness that was observed to have subcorneal opacity or one of several symptoms such as excessive tearing, soreness, eyelid swelling and preauricular lymphadenopathy with tenderness as observed by an ophthalmologist.

Based on the Korean National Disaster Level and Influenza-Like Illness surveillance, the study period was divided into three phases: 2004–2008 as ‘pre-pandemic,’ 2009 as ‘pandemic,’ and 2010 as ‘post-pandemic’ [4]. According to this classification, we assessed the change in the weekly number of EKC cases for each of the three phases. For the pre-pandemic period we estimated the mean weekly number of cases (and 95% confidence interval) of EKC reported. After separating the total number of EKC cases into the epidemic season (weeks 21 to 44) and non-epidemic season (week 0 to 20 and 45 to 52), we investigated whether there was significant variation among the pre-pandemic, pandemic and post-pandemic periods for each season. For assessing the decrease of EKC patients during influenza pandemic, we performed t-test between mean of total EKC numbers in pre-pandemic period and those in pandemic period. As the same way, we assessed the increase of EKC patients after influenza pandemic by using a t-test between pre-pandemic and post-pandemic period. These tests were performed on the assumption that annual EKC epidemic is independent each other. Patients were grouped into ten year age brackets, and analyses carried out with a view to identifying those age groups exhibiting the greatest overall change. All analyses were carried out with the use of STATA 10.0.

## Results

The number of reported EKC cases from 2004 to 2010 is shown in Table 1. The mean number of total EKC patients during the pre-pandemic period was 54,711 (95% Confidence Interval [CI], 45,499 to 64,344). By comparison with pre-pandemic period, the total number of EKC patients during pandemic period showed a significant decrease of 44.9% (t value = −7.23, p = 0.002) and those during the post-pandemic period increased to 113.0% (t value = 2.10, p = 0.104) (Table 2).

**Table 1:** EKC* cases reported from 2004 to 2010 according to age group.

	Number of EKC cases						
	2004	2005	2006	2007	2008	2009	2010
0–9yr	10,329	9,937	9,422	10,101	6,997	5,558	12,689
10–19yr	12,379	10,678	14,871	19,242	13,365	5,406	13,435
20–29yr	7,725	6,700	6,702	6,503	4,420	3,792	6,407
30–39yr	10,601	9,873	9,452	9,051	6,066	5,109	9,492
40–49yr	6,597	6,424	7,017	7,300	4,695	3,720	7,184
50–59yr	4,701	4,830	4,701	4,854	3,522	3,225	6,144
60yr or more	4,950	5,687	5,282	5,424	3,471	3,349	6,494
All ages	57,282	54,129	57,447	62,475	42,222	30,159	61,845

*Epidemic keratoconjunctivitis.

**Table 2:** Comparison of EKC patients among pre-pandemic, pandemic and post-pandemic periods.

Age	Weeks*	Periods†			t-value of t-test between pre-pandemic & pandemic‡	t-value of t-test between pre & post-pandemic‡
		Pre-pandemic cases mean [CI]	Pandemic cases	Post-pandemic cases		
Total age	Total	54711 [45282–64140]	30159	61845	t value = −7.23 (p = 0.002)	t value = 2.10 (p = 0.104)
	Epidemic	36608 [27092–46124]	17188	43095	t value = −5.67 (p = 0.005)	t value = 1.89 (p = 0.131)
	Non-epidemic	18103 [15415–20791]	12971	18750	t value = −5.30 (p = 0.006)	t value = 0.67 (p = 0.541)
0–9yr	Total	9357 [7667–11047]	5558	12689	t value = −6.24 (p = 0.003)	t value = 5.47 (p = 0.005)
	Epidemic	6223 [4859–7588]	3243	9173	t value = −6.06 (p = 0.004)	t value = 6.02 (p = 0.004)
	Non-epidemic	3134 [2590–3678]	2315	3516	t value = −4.18 (p = 0.014)	t value = 1.95 (p = 0.123)
10–19yr	Total	14107 [10072–18142]	5406	13435	t value = −5.99 (p = 0.004)	t value = −0.46 (p = 0.668)
	Epidemic	10943 [6425–15461]	3426	9869	t value = −4.62 (p = 0.010)	t value = −0.66 (p = 0.545)
	Non-epidemic	3164 [2528–3800]	1980	3566	t value = −5.17 (p = 0.007)	t value = 1.75 (p = 0.154)
20–29yr	Total	6410 [4906–7914]	3792	6407	t value = −4.83 (p = 0.008)	t value = −0.01 (p = 0.996)
	Epidemic	4071 [2950–5192]	2194	4516	t value = −4.65 (p = 0.010)	t value = 1.10 (p = 0.332)
	Non-epidemic	2339 [1827–2852]	1598	1891	t value = −4.02 (p = 0.016)	t value = −2.43 (p = 0.072)
30–39yr	Total	9009 [6845–11172]	5109	9492	t value = −5.01 (p = 0.008)	t value = 0.62 (p = 0.569)
	Epidemic	5590 [4073–7107]	2854	6627	t value = −5.01 (p = 0.007)	t value = 1.90 (p = 0.131)
	Non-epidemic	3419 [2609–4229]	2255	2865	t value = −3.99 (p = 0.016)	t value = −1.90 (p = 0.131)
40–49yr	Total	6407 [5144–7669]	3720	7184	t value = −5.91 (p = 0.004)	t value = 1.71 (p = 0.163)
	Epidemic	4035 [2749–5322]	1969	4792	t value = −4.46 (p = 0.011)	t value = 1.63 (p = 0.178)
	Non-epidemic	2371 [2004–2738]	1751	2392	t value = −4.69 (p = 0.009)	t value = 0.16 (p = 0.883)
50–59yr	Total	4522 [3822–5221]	3225	6144	t value = −5.15 (p = 0.007)	t value = 6.44 (p = 0.003)
	Epidemic	2694 [2042–3346]	1671	3897	t value = −4.36 (p = 0.012)	t value = 5.12 (p = 0.007)
	Non-epidemic	1827 [1585–2071]	1554	2247	t value = −3.11 (p = 0.036)	t value = 4.79 (p = 0.009)
≥60yr	Total	4963 [3876–6050]	3349	6494	t value = −4.12 (p = 0.015)	t value = 3.91 (p = 0.174)
	Epidemic	3115 [2220–4009]	1831	4221	t value = −3.98 (p = 0.016)	t value = 3.43 (p = 0.026)
	Non-epidemic	1848 [1608–2088]	1518	2273	t value = −3.82 (p = 0.019)	t value = 4.92 (p = 0.008)

*Total, epidemic, and non-epidemic seasons are weeks 1 to 52, weeks 21 to 44, and weeks 1 to 20 and 45 to 52, respectively.

†Pre-pandemic mean cases from 2004 to 2008, Pandemic cases in 2009, Post-pandemic cases in 2010.

‡Degrees of freedom of all values  =  4.

**Figure 1:**
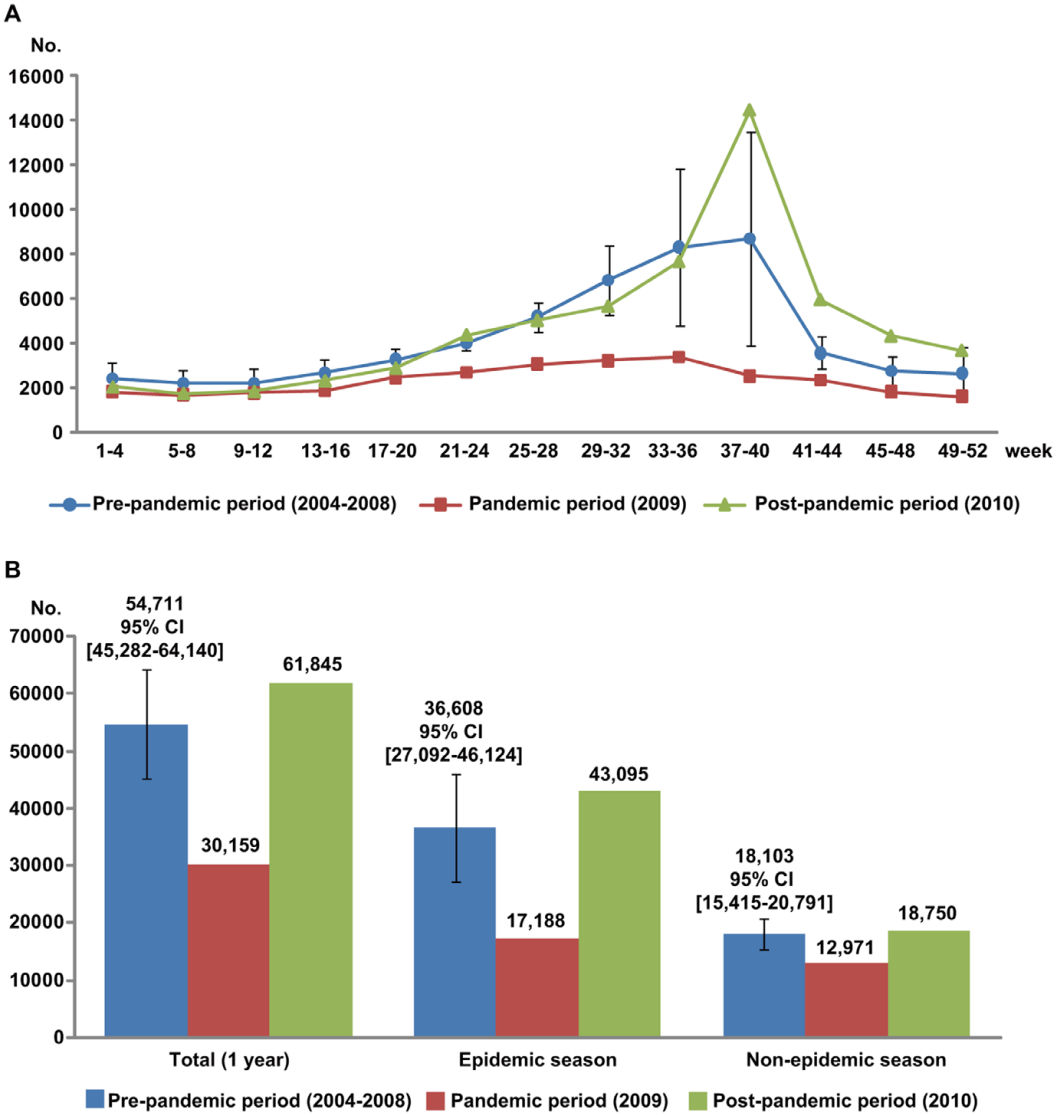
Comparison of the EKC incidence among pre-pandemic, pandemic and post-pandemic periods. A. Trends of the number of patients on a monthly basis. B. EKC incidence according to total, epidemic and non-epidemic season.

With respect to changes in the weekly EKC incidence, while an increase was observed from the 21st through to the 44th week (epidemic season) during both the pre- and post-pandemic periods, there was no epidemic peak during the pandemic period (Figure 1). In addition, there was also a significant decrease in the number of EKC patients during pandemic period in both the epidemic (t value = −5.67, p = 0.005) and non-epidemic seasons (t value = −5.30, p = 0.006) compared to the pre-pandemic period (Table 2). Moreover this result was more prominent in the epidemic season (from 36,608 to 17,188, 53.0% decrease) than in the non-epidemic season (from 18,103 to 12,971, 28.3% decrease).

**Figure 2:**
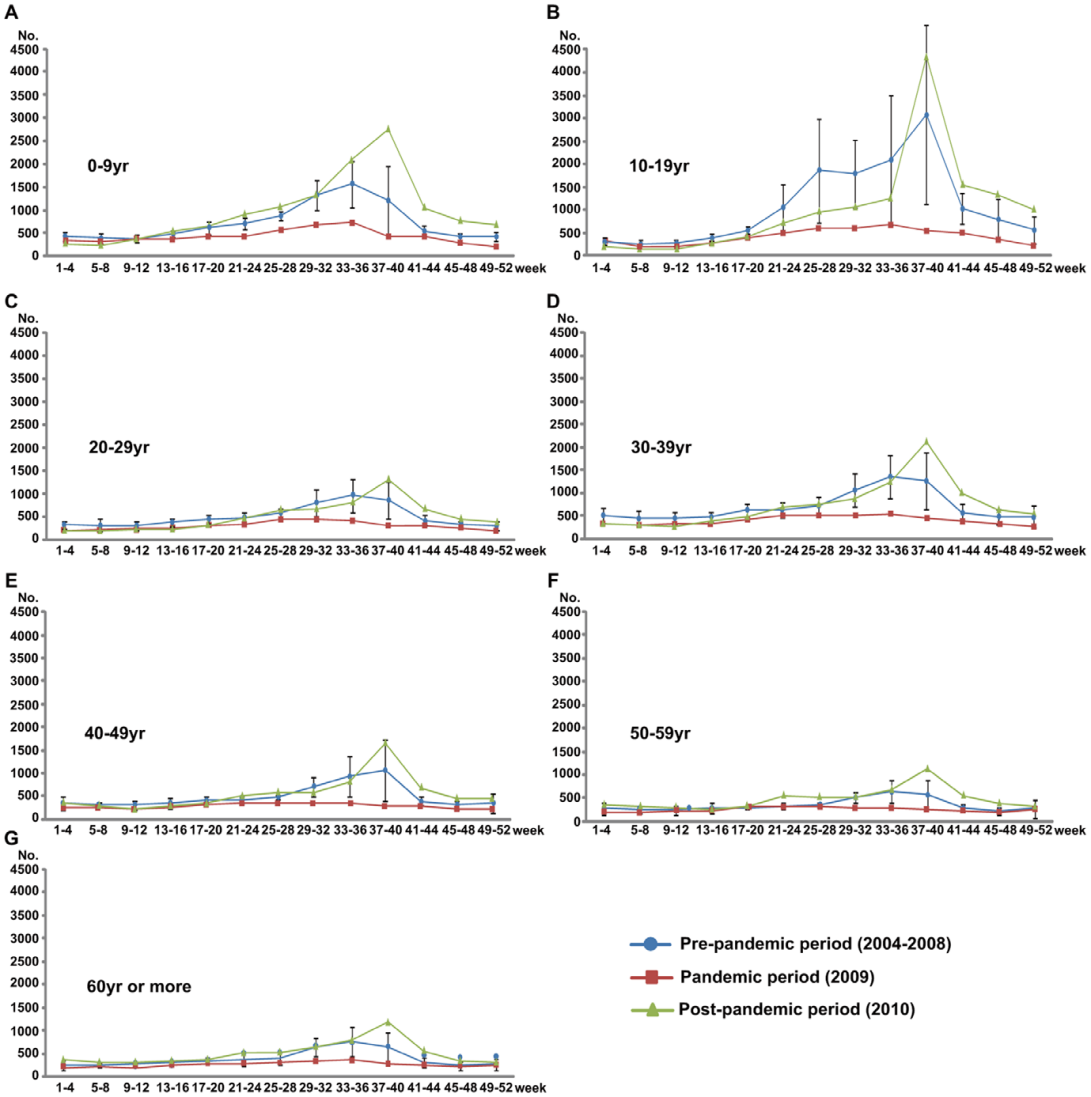
EKC cases among pre-pandemic, pandemic and post-pandemic periods according to age group.

Comparing the pre-pandemic and pandemic periods by age group, we found there to be a significant decrease in the number of EKC patients for all age groups (Figure 2, Table 2). This finding was most evident in the teenage group (decreased of 62%) compared to the other age groups (decreases of 29 to 44%)

## Discussion

This study attempted to evaluate the effect of the H1N1pandemic influenza on the incidence of epidemic keratoconjunctivitis (EKC) and hygiene behavior in Korea. Although we did not carry out a questionnaire-based study to investigate whether the public complied with recommended hygiene behavior such as hand washing, we utilized the incidence of EKC as an indirect indicator of hand washing on the assumption that hand washing is one of the most effective ways to prevent EKC infection [15]–[18].

Regarding the absence of an EKC epidemic peak during the pandemic period, it is assumed that the influenza pandemic helped to reinforce preventive behavior. This result is supported by data from a cross-sectional web-based survey of 75,000 Korean middle and high school students which reported on the percentage of students performing frequent hand washing with soap before a meal and after using the toilet; values increased dramatically to 56.5% and 72.3% respectively in October 2009 (peak pandemic season) compared to 32.8% and 47.9% in October 2008 (pre-pandemic season) [19]. However, considering that the number of EKC patients increased again just after the pandemic period, we postulate that the positive hygiene effect was maintained only during the pandemic period. These results are consistent with data suggesting that preventive behavior declined as the frequency of reports of influenza-related deaths or the intensity of influenza spread also declined [11], [14].

In addition, the number of EKC patients during the pandemic period compared to the pre-pandemic period was significantly reduced in the teenage group. The reason for this is that wide publicity and education encouraging preventive behavior may have been more effective in this age group. We recommend that health authorities may need to intensify and sustain hygiene messages to the public throughout a pandemic, especially with respect to teenagers because their incidence of EKC is returned to its high levels in the post-epidemic period.

As the influenza virus is known to compete with other respiratory viruses, the influenza pandemic could have suppressed an adenovirus epidemic, this being the main cause of EKC. However, the typical epidemic season for EKC is different from that for influenza. In North East Asia, while influenza activity tends to peak in winter or spring, seasonal epidemics of adenoviral conjunctivitis occur in the summer in association with higher temperatures and humidity [20], [21]. Therefore, an adenovirus epidemic would not have been influenced by the pandemic influenza.

We have demonstrated an association between pandemic influenza and hygiene behavior by analyzing the incidence of another infectious disease (EKC) over several years.

Our study has several limitations. First, because it had a cross-sectional design format, we cannot definitively infer a causal relationship between the increase of hand washing and the decrease of EKC. Second, the diagnosis of EKC is based on clinical symptoms, so it is possible that some forms of non-infectious conjunctivitis which are not related to hand washing, such as allergic conjunctivitis, were included in our data. However, such cases would only contribute to a very small proportion of the total number of cases of EKC. The reason for this is that epidemic season of EKC is different from that of allergic conjunctivitis and EKC has more severe symptoms that could discriminate from allergic conjunctivitis. Lastly, given the lack of specific sociodemographic informations such as sex and detailed age, we couldn't analyze the epidemic trends according to those.

In conclusion, we postulate that hygiene behaviors improved during the pandemic period, which lead to a reduction in the number of EKC cases during this period. However, this behavioral change did not persist into the post-pandemic period in Korea. This relationship was most marked in teenagers. WHO and Public Health Service of governments should do to sustain hygiene behaviors in the post-pandemic period.
